# Analysis of Acrolein Exposure Induced Pulmonary Response in Seven Inbred Mouse Strains and Human Primary Bronchial Epithelial Cells Cultured at Air-Liquid Interface

**DOI:** 10.1155/2020/3259723

**Published:** 2020-10-08

**Authors:** Gunnar Johanson, Aishwarya Mishra Dwivedi, Lena Ernstgård, Lena Palmberg, Koustav Ganguly, Lung Chi Chen, Karen Galdanes, Terry Gordon, Swapna Upadhyay

**Affiliations:** ^1^Integrative Toxicology, Institute of Environmental Medicine, Karolinska Institute, Stockholm, Sweden; ^2^Department of Environmental Medicine, NYU, School of Medicine, USA

## Abstract

**Background:**

Acrolein is a major component of environmental pollutants, cigarette smoke, and is also formed by heating cooking oil. We evaluated the interstrain variability of response to subchronic inhalation exposure to acrolein among inbred mouse strains for inflammation, oxidative stress, and tissue injury responses. Furthermore, we studied the response to acrolein vapor in the lung mucosa model using human primary bronchial epithelial cells (PBEC) cultured at an air-liquid interface (ALI) to evaluate the findings of mouse studies.

**Methods:**

Female 129S1/SvlmJ, A/J, BALB/cByJ, C3H/HeJ, C57BL/6J, DBA/2J, and FVB/NJ mice were exposed to 1 part per million (ppm) acrolein or filtered air for 11 weeks. Total cell counts and protein concentrations were measured in bronchoalveolar lavage (BAL) fluid to assess airway inflammation and membrane integrity. PBEC-ALI models were exposed to acrolein vapor (0.1 and 0.2 ppm) for 30 minutes. Gene expression of proinflammatory, oxidative stress, and tissue injury-repair markers was assessed (cut off: ≥2 folds; *p* < 0.05) in the lung models.

**Results:**

Total BAL cell numbers and protein concentrations remained unchanged following acrolein exposure in all mouse strains. BALB/cByJ, C57BL/6J, and 129S1/SvlmJ strains were the most affected with an increased expression of proinflammatory, oxidative stress, and/or tissue injury markers. DBA/2J, C3H/HeJ, A/J, and FVB/NJ were affected to a lesser extent. Both matrix metalloproteinase 9 (*Mmp9*) and tissue inhibitor of metalloproteinase 1 (*Timp1*) were upregulated in the strains DBA/2J, C3H/HeJ, and FVB/NJ indicating altered protease/antiprotease balance. Upregulation of lung interleukin- (*IL*-) *17b* transcript in the susceptible strains led us to investigate the IL-17 pathway genes in the PBEC-ALI model. Acrolein exposure resulted in an increased expression of *IL-17A*, *C*, and *D*; *IL-1B*; *IL-22*; and *RAR-related orphan receptor A* in the PBEC-ALI model.

**Conclusion:**

The interstrain differences in response to subchronic acrolein exposure in mouse suggest a genetic predisposition. Altered expression of IL-17 pathway genes following acrolein exposure in the PBEC-ALI models indicates that it has a central role in chemical irritant toxicity. The findings also indicate that genetically determined differences in IL-17 signaling pathway genes in the different mouse strains may explain their susceptibility to different chemical irritants.

## 1. Introduction

Acrolein (2-propenal) is a highly volatile and reactive *α*-*β*-unsaturated aldehyde that is primarily used as an intermediate in chemical manufacturing [1]. It is also produced during endogenous oxidative processes and is a major bioactive component of environmental pollutants such as automobile exhaust, biomass fuel, burning of wood and plastics, and smog. Acroleins are also formed by heating cooking oil and fat (above 300°C) during domestic cooking [2]. Acrolein is an important constituent of mainstream cigarette smoke with concentrations about 90 ppm [3]. The major effects of inhalation exposure to acrolein in humans and animals result in eye, nose, and throat irritation. Nasal irritation, activation of sensory nerves in the nasal mucosa, increaseexposure (0.31-1.7 ppm) in rodent studies [2].

Several *in vivo* studies have demonstrated that acute exposure to 0.2 to 6 ppm acrolein causes pulmonary edema, stimulates sensory nerves and airway cell proliferation, and diminishes pulmonary defenses against bacterial and viral infection [4]. Leikauf et al. [5] performed a study exposing 40 inbred mouse strains to 10 ppm acrolein to induce acute lung injury [1]. The study demonstrated remarkable variation in survival times among the mouse strains (<20 h: 13 strains; 20-30 h: 20 strains; >30 h: 7 strains) following acrolein-induced acute lung injury. The study was extended to identify genes that may render susceptibility or resistance to acrolein-induced acute lung injury by comparing the polar strains. Acute lung injury is a sporadic event, and case-based observation suggests great variability in individual susceptibility; i.e., individuals with the same severity score can have markedly different clinical outcomes thereby suggesting a high degree of genetic predisposition [5].

Borchers et al. [6] examined the effect of 0.01-100 nM acrolein on mucin gene expression in cultured human airway epithelial cells. Increased mucin (*MUC5AC*) transcript levels following a 4 h exposure to acrolein in cultured human airway epithelial cells were reported. Inflammation, oxidative stress, and tissue destruction are considered as primary adverse outcome pathways of most chronic lung diseases (McGuinness and Sapey [7]). Therefore, in this study, we sought to investigate variability in pulmonary response to subchronic acrolein exposure among seven inbred mouse strains measured as oxidative stress, proinflammatory response, and tissue injury. The study was aimed at investigating acrolein as an irritant model substance with a plausible genetic susceptibility contribution. The survival time of the seven inbred mouse strains selected for our study was spread across the survival times of the 40 inbred mouse strains (female) observed by Leikauf et al. [5]. together with easy availability and common use in respiratory research. Based on our findings of altered lung interleukin-17B (*IL-17B*) transcript expression in acrolein-exposed mice, we further investigated the IL-17 pathway genes in the human primary bronchial epithelial cells (PBEC) cultured at an air-liquid interface (ALI) following 0.1 and 0.2 parts per million (ppm) acrolein exposure.

## 2. Methods

### 2.1. Animals

Female 129S1/SvlmJ (stock number 002448), A/J (stock number 000646), BALB/cByJ (stock number 001026), C3H/HeJ (stock number 000659), C57BL/6J (stock number 000664), DBA/2J (stock number 000671), and FVB/NJ (stock number 001800) mice (age: 10 weeks) were obtained from the Jackson Laboratory (Bar Harbor, ME, USA), housed under specific pathogen-free conditions at the animal facility of the New York University, School of Medicine (NY, USA), and quarantined for at least 2 weeks before study initiation. Food and water were provided *ad libitum* except during exposure. The study was approved by the Institutional Animal Care and Use Committee, New York University, School of Medicine (NY, USA). We used only female mice for our study to minimize the animal numbers and for comparative analyses with other studies [5].

### 2.2. Mouse Procedures

#### 2.2.1. Acrolein Exposure

Mice (*n* = 5/group/strain) were exposed to filtered air or acrolein vapor *via* whole-body exposure in 1.3 M^3^ stainless steel inhalation exposure chambers for 6 h/day, 4-5 days/week for a total of 11 weeks. Acrolein vapor was generated by passing charcoal and HEPA-filtered air over acrolein (Sigma Chemical Co.) in a glass flask. The chamber concentration of acrolein was monitored on an hourly basis on each exposure day (target concentration of 1 ppm) with a Miran 1A single-beam infrared spectrometer (Foxboro Analytical, Foxboro, MA). The actual chamber concentration was 1.03 ± 0.03 ppm (mean ± SD). The 1 ppm dosage was a high dose to start with and nonlethal for a repeated exposure study, and in addition, it was an order of magnitude lower than that used in acute lung injury exposure studies.

#### 2.2.2. Bronchoalveolar Lavage (BAL)

At 24 h after the final exposure, mice were euthanized by intraperitoneal injections of ketamine HCl (100 mg/kg; Vetalar, Fort Dodge Laboratories, Fort Dodge, IA) and sodium pentobarbital (175 mg/kg; Sleepaway, Fort Dodge Laboratories), and the posterior abdominal aorta was severed. The lungs of each mouse were lavaged two times with 1.2 ml of Dulbecco's phosphate-buffered saline without Ca or Mg (pH 7.2-7.4, 37°C; GibcoBRL, Life Technologies, Grand Island, NY). The recovered BAL fluid was immediately placed on ice (4°C). The lavage fluid was then centrifuged (500 x g, 8 min, 4°C), and the supernatant was pipetted off. The total protein concentration in the supernatant was measured using an assay kit that follows the method of Bradford (1976) [8] (bovine serum albumin standard, 550 nm; Pierce, Rockford, IL). The total protein concentration in BAL fluid was used as an indicator of changes in lung permeability and injury [9]. Total cell counts were determined with a hemacytometer.

#### 2.2.3. Lung Transcript Expression Analysis

Total lung RNA was isolated using the RNeasykit (catalog number 74104; Qiagen, Hilden, Germany) according to the manufacturer's instruction, and the concentration was quantified using Nanodrop 2100 (ThermoFisher, USA). The extracted total RNA was stored at -80°C until further use (supplementary file). Transcripts assayed using the qRT-PCR technique included beta actin (*Actb*), chemokine (C-X-C motif) ligand 1 (*Cxcl1*), *Cxcl2*, glutathione peroxidase 1 and 3 (*Gpx1*, *Gpx3*), heme oxygenase 1 (*Hmox1*), *interleukin-6 (IL-6)*, *IL-17B*, matrix metalloproteinase 9 (*Mmp9*), nuclear factor of kappa light polypeptide gene enhancer in B cells 1, p105 (*Nfkb1*), *superoxide dismutase 3*, *extracellular* (*Sod3*), tissue inhibitor of metalloproteinase 1 (*Timp1*), and tumor necrosis factor (*Tnf*). Beta actin (*Actb*) was used as the reference control. Forward and reverse primer sequences are provided in supplementary Table S1.

## 3. Air-Liquid Interface (ALI) Model Development

The human airway mucosa model was developed using PBEC from 3 individual (*N* = 3) donors and cultured at ALI as previously described [10–12]. All procedures performed in this study were in accordance with the approval of the Ethics Committee of Karolinska Institutet, Stockholm. The PBEC that we have used in this study are well characterized and used in several previous studies [13, 14]. PBEC from passages 3 or 4 were grown under submerged conditions in Petri dishes and were cultured up to 80% confluency at 37°C in a humidified atmosphere of 5% CO_2_ using a serum-free keratinocyte medium (KSFM; Gibco, USA) supplemented with 5 ng/ml epidermal growth factor (EGF; Gibco, USA), 50 *μ*g/ml bovine pituitary extract (BPE; Gibco, USA), and 20 U/ml pen/strep (Gibco, USA). The medium was changed every second day. Cells from the Petri dishes were trypsinized, resuspended, and seeded on precoated 0.4 *μ*m semiporous transwell inserts (BD Falcon™, USA), placed in twelve well plates at a density of 0.1 million cells/well. The inserts were maintained in submerged conditions with 1 ml complete KSFM on both (inner and outer) sides of inserts. Once cells reached 90% confluence, the inserts were turned upside down and put in a sterile Petri dish to add MRC-5 cells (Medical Research Council cell strain 5, fibroblasts derived from human lung tissue) in a complete Dulbecco's Modified Eagle's Medium to the outer or basal side of the insert membrane [10]. After 30 minutes of incubation, the inserts were again placed in the plate with 1 ml of complete KSFM per well, on both inner (apical) and outside (basal side) of the insert. The next day, the models were air-lifted by removing the medium and adding 870 *μ*l coculture medium (i.e., complete KSFM with 6 *μ*g/ml CaCl_2_ in double-distilled water (ddH_2_O), 15 ng/ml ethanolamine in ddH_2_O, and 10-5 M retinoic acid) basal or outside the insert. The cells were incubated at 37°C with 5% CO_2_ for at least two weeks till they began to differentiate into different cell types: mucus-producing cells, ciliated cells and club cells, and basal cells. Three technical replicates (*n* = 3/donor/acrolein concentration) were developed from each donor and were exposed for 30 minutes to different concentrations of acrolein vapor separately (see below). Finally, the expression of genes within the IL-17 pathway was analyzed at 24 h postexposure to acrolein.

### 3.1. Acrolein Exposure of the ALI Model

Three technical replicates developed from each donor were exposed for 30 minutes to clean air (sham), 0.05, 0.1, 0.2, and 0.5 ppm (0, 0.1, 0.2, 0.5, and 1.1 mg/m^3^) acrolein using our in-house developed exposure systems as described in previous studies [10, 12]. The actual acrolein concentrations in the ALI chamber air were monitored by gas chromatography (five times during 30 minutes), as described previously [10], and were on average 17-25% higher than the target concentrations. Cell viability of PBEC was tested at 24 h after exposure by trypan blue staining (200 *μ*l of 1 : 1 in PBS diluted 0.4% trypan blue solution for 1 minute), washing by PBS, and followed by bright-field microscopy [10]. At 0.5 ppm, cell viability was 70% ± 5% (mean ± SD) [10]. Since none of the other doses showed any cytotoxicity [2], further analysis was carried out in 0.1 and 0.2 ppm acrolein-exposed samples compared to sham. Based on the findings of altered *Il-17B* regulation in the mouse lungs following subchronic acrolein exposure, we explored the IL-17 pathway in acrolein-exposed PBEC. Transcript expression of *IL-17A*, *IL-17B*, *IL-17C*, *IL-17D*, *IL-17E*, *IL-1B*, *IL-22*, RAR-related orphan receptor A (*RORA*), and signal transducer and activator of transcription 3 (*STAT3*) was analyzed 24 h after the 30 min exposure to clean air (sham), 0.1 and 0.2 ppm acrolein. Forward and reverse primer sequences are provided in supplementary table S1.

### 3.2. Statistical Analyses

The results are generally expressed as medians and interquartile ranges (25th-75th percentiles) normalized with its own sham (control). For the *in vivo* study, the Friedman test was used to analyze differences between sham (clean air: control) and acrolein-exposed animals of each strain. Additionally, the interstrain differences of *in vivo* studies were compared by Kruskal-Wallis followed by Mann-Whitney. Similarly, for *in vitro* study, the Friedman test was used to analyze differences between sham (clean air: control) and different concentrations of acrolein used to expose the *in vitro* ALI model, followed by the Wilcoxon signed-rank *t* test as a post hoc test. A *p* value < 0.05 was considered as statistically significant. All data were analyzed using the STATISTICA13 software (StatSoft, Inc., Uppsala, Sweden). The statistical analysis of transcript expression was carried out using the dCT values.

## 4. Results

### 4.1. BAL Analysis

Total BAL cell counts and protein concentrations showed no changes in the acrolein-exposed mice compared to their respective strain control exposure (supplementary Table S2 and Table S3).

### 4.2. Transcript Expression in Mouse Lungs

**Figure 1:**
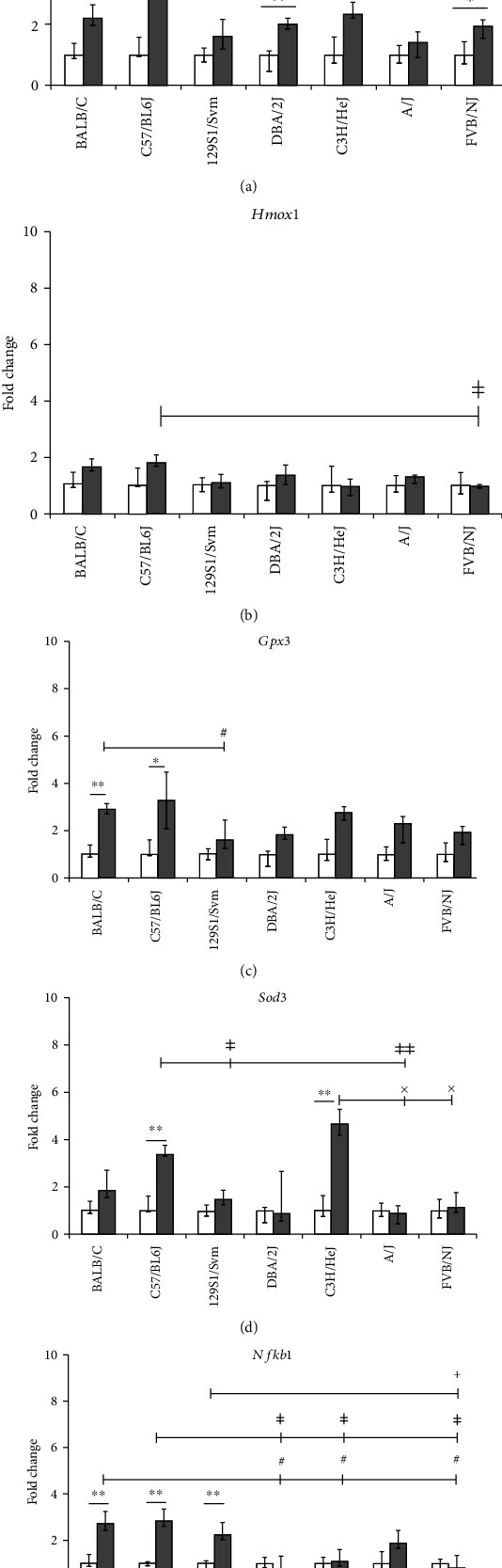
Lung transcript analysis of oxidative stress markers in seven mouse strains exposed to clean air (sham) or acrolein (1 ppm, 6 hours/day, 4-5 days/week for 11 weeks): (a) glutathione peroxidase 1 (*Gpx1*); (b) glutathione peroxidase 3 (*Gpx3*); (c) superoxide dismutase 3, extracellular (*Sod3*); (d) heme oxygenase 1 (*Hmox1*); (e) nuclear factor of kappa light polypeptide gene enhancer in B cells 1, p105 (*Nfkb1*). Beta actin (*Actb*) served as the housekeeping control gene. Data are presented as an expression in the exposed group relative to age and strain-matched sham-exposed group (median and 25th-75th percentiles; *n* = 5 mice/strain/stage); white bars: sham, gray bars: acrolein exposed. ^∗^*p* value < 0.05 and ^∗∗^*p* value < 0.01; sham vs. exposed group of each strain. ^#^*p* value < 0.05, exposed BALB/cByJ vs. other exposed strains like DBA/2J, C3H/HeJ, and A/J. ^ǂ^*p* value < 0.05 and ^ǂǂ^*p* vaue < 0.01; exposed C57BL/6J vs. exposed strains like DBA/2J, C3H/HeJ, and FVB/NJ. ^+^*p* value < 0.05, exposed 129S1/SvlmJ vs. exposed FVB/NJ. ^x^*p* value < 0.05, exposed C3H/HeJ vs. exposed A/J vs. FVB/NJ.

**Figure 2:**
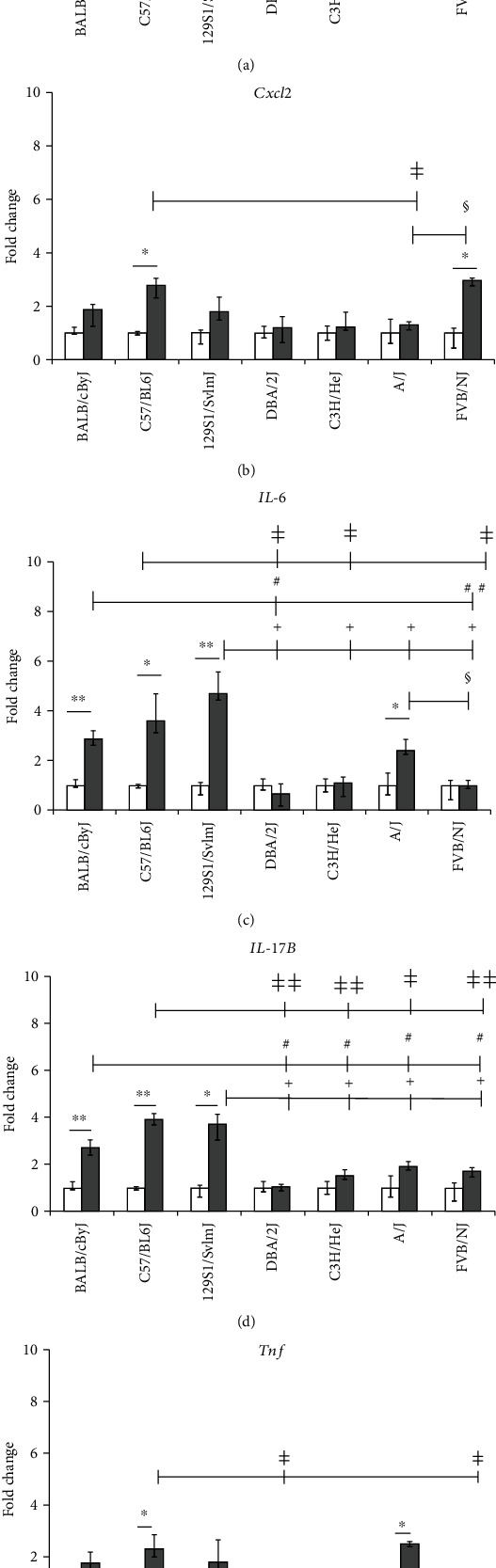
Lung transcript analysis of proinflammatory markers in seven mouse strains exposed to clean air (sham) or acrolein (1 ppm, 6 hours/day, 4-5 days/week, 11 weeks): (a) chemokine (*C-X-C motif*) ligand 1 (*Cxcl1*); (b) chemokine (C-X-C motif) ligand 2 (*Cxcl2*); (c) *interleukin-6* (*IL-6*); (d) *interleukin-17B* (*IL-17B*); (e) tumor necrosis factor (*Tnf*). Beta actin (*Actb*) served as the control. Data are presented as an expression in the exposed group relative to age and the strain-matched sham-exposed group (median and 25th-75th percentiles; *n* = 5 mice/strain/stage). Mouse strains: 129S1/SvlmJ, A/J, BALB/cByJ, C3H/HeJ, C57BL/6J, DBA/2J, and FVB/NJ; white bars: sham, gray bars: acrolein exposed. ^∗^*p* value < 0.05 and ^∗∗^*p* < 0.01; sham vs. exposed group of each strain. ^#^*p* < 0.05 and ^##^*p* < 0.01; exposed BALB/cByJ vs. other exposed strains like DBA/2J, C3H/HeJ, and A/J. ^ǂ^*p* value < 0.05 and ^ǂǂ^*p* value < 0.01; exposed C57BL/6J vs. exposed strains like DBA/2J, C3H/HeJ, and FVB/NJ. ^+^*p* value < 0.05, exposed 129S1/SvlmJ vs. exposed strains DBA/2J, C3H/HeJ, A/J, and FVB/NJ. ^♦^*p* value < 0.05, exposed DBA/2J vs. exposed. ^§^*p* value < 0.05, exposed A/J vs. exposed FVB/NJ.

The BALB/cByJ, C57BL/6J, and 129S1/SvlmJ mouse strains exhibited the highest increase in mRNA expression levels for both oxidative stress (*Gpx1*, *Gpx3*, Nfkb1, and *Sod3*; 2-5 fold; Figures 1(a)–1(e)) and proinflammatory markers (*Cxcl1*, *Cxcl2*, *IL-6*, *IL-17B*, and *Tnf*: ~2- to 4-fold, Figures 2(a)–2(e)). High oxidative stress response (*Gpx*1: ~2-fold increased and *Sod3*: >4-fold increased; Figures 1(a) and 1(e)) was also detected in C3H/HeJ compared to a comparatively low oxidative stress response in DBA/2J and FVB/NJ strains (Figure 1(a)). Interestingly, the oxidative stress marker *Ho1* did not show any significant alteration in any of the investigated seven inbred mouse strains exposed to subchronic doses of acrolein (Figure 1(d)). The A/J and FVB/NJ strains exhibited a medium proinflammatory response as observed by ~2-fold increase of *Tnf*, *Cxcl1*, *Cxcl2*, and *IL-6* transcripts (Figures 2(a)–2(e)). C3H/HeJ and DBA/2J mice did not show any alteration in these proinflammatory markers.

**Figure 3:**
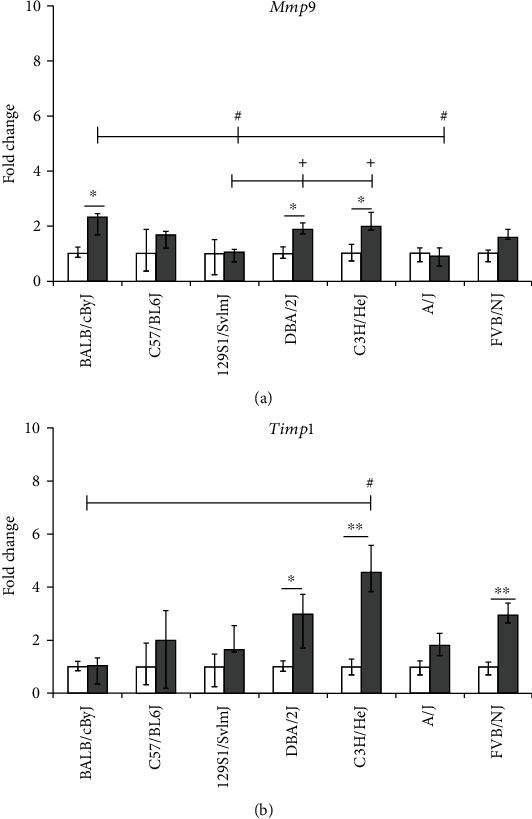
Lung transcript analysis of tissue injury markers in seven mouse strains exposed to clean air (sham) or acrolein (1 ppm, 6 hours/day, 4-5 days/week, 11 weeks): (a) matrix metallopeptidase 9 (*Mmp9*); (b) tissue inhibitor of metalloproteinase 1 (*Timp1*). Beta actin (*Actb*) served as the control. Data are presented as an expression in the exposed group relative to age and the strain-matched sham-exposed group (median and 25th-75th percentiles; *n* = 5 mice/strain/stage). Mouse strains: 129S1/SvlmJ, A/J, BALB/cByJ, C3H/HeJ, C57BL/6J, DBA/2J, and FVB/NJ; white bars: sham; gray bars: acrolein exposed. ^∗^*p* value < 0.05, ^∗∗^*p* value < 0.01: sham vs. exposed group of each strain. ^#^*p* value < 0.05, exposed BALB/cByJ vs. exposed 129S1/SvlmJ, C3H/HeJ, and A/J. ^+^*p* value < 0.05, exposed 129S1/SvlmJ vs. exposed DBA/2J and C3H/HeJ.

To determine the effect of acrolein exposure on proteinase and antiproteinase homeostasis, measurements of the expression of *Mmp9* and *Timp1* were performed (Figures 3(a) and 3(b)). *Mmp9* was increased in BALB/cByJ, C3H/HeJ, and DBA/2J by ~2-fold (Figure 3(a)), and a corresponding >3-fold increase in *Timp1* was detected in the lungs of C3H/HeJ and DBA/2J but not in BALB/cByJ mice (Figure 3(b)). *Timp1* expression was also increased by ~2-fold in FVB/NJ mice. Basal lung transcript levels of some selected oxidative stress, proinflammatory, and extracellular matrix markers among the seven inbred mouse strains are shown in Table 1. None of the genes exhibited any statistically significant difference in their transcript expression among the sham-exposed groups. The interstrain difference in transcript expression of the investigated genes following acrolein exposure is shown in Figures 1–3.

### 4.3. Analysis of the IL-17 Pathway

**Figure 4:**
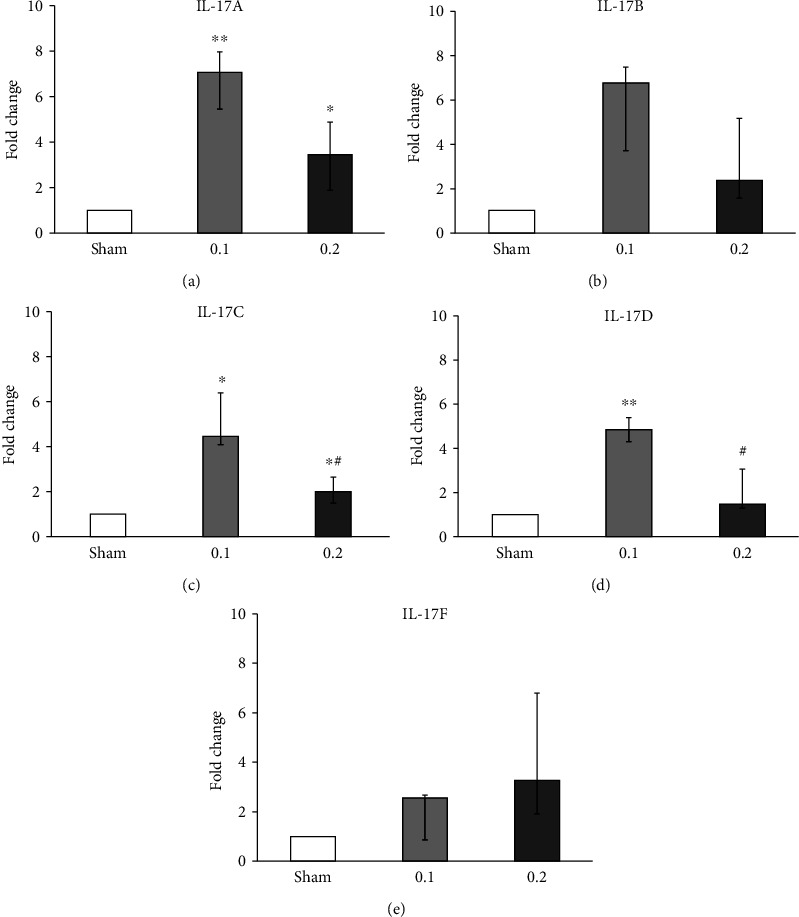
Transcript analysis of IL-17 family members (IL-17A, B, C, D, and F) in primary bronchial epithelial cells (PBEC) cultured at an air-liquid interface (ALI) condition after 24 h incubation following a 30-minute exposure to clean air (sham), 0.1 or 0.2 ppm acrolein vapor: (a) interleukin-17A (*IL-17A*); (b) interleukin-17B (*IL-17B*); (c) interleukin-17C (*IL-17C*); (d) interleukin-17D (*IL-17D*); (e) interleukin-17F (*IL-17F*). Beta actin (*ACTB*) served as the control. Data are presented as median and 25th-75th percentiles (*N* = 3 donors and *n* = 2 replicates/donor). ^∗^*p* value < 0.05, ^∗∗^*p* value < 0.01: sham vs. exposed group; ^#^*p* value < 0.05 0.1 ppm vs. 0.2 ppm acrolein exposed. White bars: sham (clean air); light gray bars: 0.1 ppm; dark gray bars: 0.2 ppm acrolein exposed.

**Figure 5:**
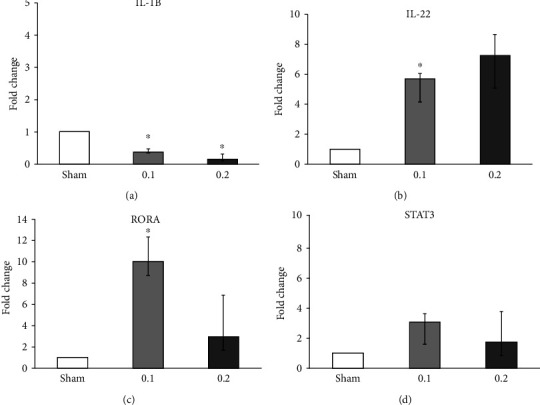
Transcript analysis of interleukin-17 (*IL-17*) pathway-related markers (*IL-1B*, *IL-22*, RORA, and STAT3) in primary bronchial epithelial cells (PBEC) cultured at an air-liquid interface (ALI) condition after 24 h of incubation following a 30-minute exposure to clean air (sham), 0.1 or 0.2 ppm acrolein vapor. Transcript expression of interleukin-1 beta (*IL-1B)*, interleukin-22 (*IL-22*), RAR-related orphan receptor A (RORA), and signal transducer and activator of transcription 3 (STAT3) was analyzed following 24 h of exposure. Data are presented as median and 25th-75th percentiles (*N* = 3 donors and *n* = 2 replicates/donor). ^∗^*p* value < 0.05; ^∗∗^*p* value < 0.01; white bars: sham (clean air); light gray bars: 0.1 ppm exposed; dark gray bars: 0.2 ppm acrolein exposed.

Significantly increased expression of *IL-17B* was detected in the BALB/cByJ, C57BL/6J, and 129S1/SvlmJ mouse strains following subchronic exposure to 1 ppm acrolein. IL-17, the signature cytokine secreted by Th17 cells, is required for host defense mechanisms. Altered regulation of IL-17-related cytokines can contribute to the pathogenesis of various inflammatory diseases [15]. Therefore, in this study, we further explored the transcript expression pattern of several key IL-17 cytokine family members (*IL-17A*, *B*, *C*, *D*, and *F*; Figures 4(a)–4(e)) 24 h postexposure to 0.1 and 0.2 ppm acrolein vapor in PBEC cultured at ALI condition. Additionally, we have also analyzed the expression of *RORA*, *STAT3*, *IL-1B*, and *IL-22* which are also considered important members of the IL-17 family.  Figure 4 exhibits the significantly increased expression of *IL-17A* (Figure 4(a)) and IL-17C (Figure 4(c)) in PBEC following exposure to both 0.1 and 0.2 ppm acroleins compared to sham. The expression of *IL-17D* was significantly upregulated after exposure to 0.1 ppm acrolein (Figure 4(d)), similar to the expression of *IL-17C* following exposure to 0.2 ppm acrolein. In general, the expression of most of the IL-17 isotypes exhibited a stronger response at 0.1 ppm acrolein compared to 0.2 ppm. Furthermore, *IL-1B* showed significantly reduced expression at both 0.1 and 0.2 ppm acroleins (Figure 5(a)). While the expression of *RORA* and *IL-22* was significantly increased at 0.1 ppm acrolein (Figures 5(b) and 5(c)), and *STAT3* expression remained unaltered at both 0.1 and 0.2 ppm acroleins (Figure 5(d)).

## 5. Discussion

Acrolein- (10 ppm) induced acute lung injury in female mice has shown that the survival time among the most polar strains varied approximately 2.5-fold from 16 h (susceptible) to 41 h (resistant). Furthermore, based on survival time, the 40 inbred strains can be broadly categorized into three groups: (i) <20 h: 13 strains, (ii) 20-30 h: 20 strains, and (iii) ≥30 h: 7 strains. These findings strongly support the genetic predisposition to acrolein-induced acute lung injury among mice. The survival time of our investigated 7 strains to acrolein-induced acute lung injury was in the following order BALB/cByJ (<20 h) < C57BL/6J < 129S1/SvlmJ<DBA/2J < C3H/HeJ < A/J (20-30 h) < FVB/NJ (>30 h) [5]. Therefore, the selected mouse strains in this project were spread across the three broad categories of survival time for acrolein-induced acute lung injury. We investigated markers of three key events of adverse outcome pathways for chronic lung diseases, namely, oxidative stress, inflammation, and tissue injury/repair in the lungs of the 7 inbred mouse strains following a 1 ppm subchronic acrolein exposure. We further attempted to rank the 7 strains based on the altered expression of these markers for susceptibility to 1 ppm subchronic acrolein exposure. The targeted acrolein exposure concentration (1 ppm) was lower compared to indoor environmental conditions (5 ppm) due to smoking, wood burning, or cooking [3, 16]. The findings of the oxidative stress, proinflammatory, and tissue injury markers have been summarized in Table 2 to identify the biologically plausible sensitivity and resistance parameters.

Our findings show that subchronic exposure to low concentrations (1 ppm) of acrolein did not cause inflammatory cell recruitment or alveolar barrier leakage as indicated by unaltered total BAL cell number and protein concentrations (Table S2 and S3). Consistent with our findings, a lack of inflammatory cell recruitment following exposure of C57BL/6J mice to acrolein (5 ppm, 6 h/day for up to 3 days) was also reported by Kashara et al. [3]. The authors hypothesized that acrolein-mediated reductions in cytokine production in C57BL/6J mice may be related to decreased antibacterial or antiviral host defense which may in turn contribute to increased susceptibility to infection or chronic lung diseases [3].

Based on the observed altered expression of the analyzed genes, the BALB/cByJ, C57BL/6J, and 129S1/SvlmJ inbred strains were most affected (Figures 1–3). The strains BALB/cByJ, C57BL/6J, and 129S1/SvlmJ had the lowest survival time following acute exposure to acrolein among the 40 mouse strains [5]. DBA/2J, C3H/HeJ, A/J, and FVB/NJ were also affected, but to a lesser extent (Figures 1–3). This may be attributed to the upregulation of the tissue injury repair marker *Timp1* among these strains (Figure 3).

In spite of similar basal lung transcript levels of the investigated oxidative stress, proinflammatory, and extracellular matrix marker genes in sham (control group), acrolein exposure resulted in different response patterns of those genes among the seven mouse strains (Figures 1–3). These interstrain differences in responses suggest that acrolein-induced lung injury may be driven by genetic susceptibility.

Increased lung *Nfkb1*, *Sod3*, *Gpx1*, and *Gpx3* transcript expression levels strongly support the onset of oxidative stress due to subchronic acrolein exposure (Figure 1). Several other studies have also demonstrated that reactive oxygen species (ROS) and *Tnf* can increase the activation of *Nfkb1* [17–19]. Increased *Nfkb1* lung transcripts in the acrolein-exposed BALB/cByJ, C57BL/6J, and 129S1/SvlmJ mice may cause substantial proinflammatory reactions (Figure 1(e)). The pulmonary *Nfkb1* signaling pathway serves as a critical modulator for airway hyperresponsiveness to inhaled agents [20], and *Nfkb1* is a central transcription factor for the production of numerous inflammatory cytokines (Figure 1(e)). Nfkb1 is also considered as a generic marker of toxic stress which may explain the different patterns of lung *Nfkb1* expression among the mouse strains compared to other oxidative stress markers [21]. Cheng et al. [22] demonstrated that *Nfkb1* signaling in nonimmune cells is a critical determinant for the pulmonary response to injurious stimuli and has a great impact on a number of key biological processes, including host defense mechanisms. Different groups have [20, 22] reported that the activation of *Nfkb1* in airway epithelial cells of mice leads to the increased expression of several cytokines and chemokines such as *IL-6*, *Cxcl1*, *Cxcl2*, and *IL-17*. Our findings in the lungs of subchronically acrolein-exposed mice are consistent with these studies. It is well established that Nfkb1 is involved in IL-6 production [23, 24], and therefore, our findings of simultaneously increased lung *IL-6* levels in the same mouse strains with increased lung *Nfkb1* are consistent (Figures 1 and 2). Since IL-17 can also induce IL-6 expression, the pattern of transcript expression in the lungs of acrolein-exposed mouse strains correlates (Figure 2) and supports the validity of the mouse models.

An imbalance of proteinases and antiproteinases and especially Mmp and Timp (Figures 3(a) and 3(b)) is an important feature of the pathogenesis of chronic lung diseases [25, 26], and their expression is regulated by various inflammatory markers such as *Tnf*, *IL-6*, *Cxcl1* [27, 28], and *Cxcl2*. Therefore, the findings of the present study, i.e., increased *Mmp9* along with unaltered *Timp1* transcript expression in the acrolein-exposed BALB/cByJ strain, indicate susceptibility to acrolein (Figures 3(a) and 3(b)). Thus, low levels of Timp1 can cause increased sensitivity to acrolein and other chemical irritants. However, in the more resistant strains (DBA/2J, C3H/HeJ, and FVB/NJ), both *Mmp9* and *Timp1* were upregulated suggesting a level protease-antiprotease balance to counter tissue injury (Figures 3(a) and 3(b)). Upregulation of *IL-17B* in the lungs of the more susceptible strains (i.e., BALB/cByJ, C57BL/6J, and 129S1/SvlmJ, Figure 2(d)) is an important observation. The IL-17 pathway has been recognized as a proinflammatory cytokine pathway involved in chronic airway inflammation among asthmatics [29, 30]. Data suggesting a role for IL-17 in tobacco smoke-induced lung diseases, including COPD, has also emerged [31, 32]. Acrolein is a major component of tobacco smoke. It is plausible that the increased transcript expression of *IL-17B* detected in mouse lungs may be due to the stimulation of resident CD4+ cells by proinflammatory cytokines such as IL-6, Cxcl1, Cxcl2, and Tnf as described by Wang et al. [30]. This led us to investigate the IL-17 pathway genes in more detail in the PBEC-ALI model following exposure to 0.1 and 0.2 ppm acrolein.

Increased expression of *IL-17A*, *C*, and *D* in the bronchial mucosa model following 0.1 ppm acrolein exposure (Figures 4(a)–4(e)) indicates the plausible airway inflammation that may be caused by low-dose acrolein exposures relevant for the onset of chronic lung diseases such as asthma and COPD. Increased expression of *IL-1B*, *IL-22*, and *RORAv* (Figures 5(a)–5(c)) following acrolein exposure further demonstrates IL-17 as a candidate pathway for low-dose acrolein exposure-mediated airway effects. It is plausible that IL-17 is indirectly triggered by cells damaged by acrolein and IL-17 may have a central role in the response to chemical irritants. Mechanistic studies focused on the role of IL-17 to other aldehydes will enhance our understanding in this aspect.

To summarize, our study utilized 7 inbred mouse strains (female) that are commonly used in respiratory research and demonstrated that subchronic exposure to low-dose (1 ppm) acrolein does not result in transient neutrophilia and lung injury. However, it does induce significant and strain-selective changes in the expression of several oxidative stress, proinflammatory, and tissue injury/repair markers leading to the identification of sensitive and resistant mouse strains for use in genetic susceptibility studies. Consistent findings of a simultaneous increase in lung *Nfkb1*, *Il6*, and *IL-17B* within the same acrolein-exposed mouse strains support the validity of the models. The findings also indicate that genetically determined differences in IL-17 signaling pathway genes in the different mouse strains may explain their susceptibility to different chemical irritants. On the basis of our mouse studies, we have further investigated the effects of low-dose acrolein exposure on PBEC cultured at ALI and thus identified IL-17 as a candidate pathway for low-dose acrolein-induced effects in human primary bronchial epithelial cells cultured at an air-liquid interface. Moreover, our findings implicate that low-dose acrolein exposure impairs the innate immune response in the airways which may in turn result in a predisposition to chronic lung diseases such as asthma and COPD. Therefore, it is plausible that IL-17 plays a central role in chemical irritants and it will be important to elucidate if the IL-17 pathway has a specific role in acrolein-mediated toxicity through mechanistic and functional studies.

## Figures and Tables

**Table 1:** Fold change calculation of selected oxidative stress, proinflammatory, and tissue injury/repair markers in sham (clean air) in six mouse strains compared to one of the most sensitive mouse strains (BALB/BL6J) used in this study.

Expression markers	BALB/cByJ	C57/BL6J	129S1/SVLMJ	DBA/2J	C3H/HeJ	A/J	FVB/NJ
Oxidative stress							
*Hmox1*	1	0.59	0.34	1.19	0.67	1.99	1.42
*Nfkb1*	1	0.95	1.37	0.53	1.15	0.46	1.17
Inflammation							
*Cxcl1*	1	0.79	1.29	1.08	0.89	0.57	0.75
*Cxcl2*	1	0.69	0.59	0.94	1.08	1.16	1.23
*IL-6*	1	1.32	1.66	0.74	1.09	1.18	1.28
*Tnf*	1	1.1	0.92	0.82	0.69	1.04	0.91
Tissue injury/repair							
*Mmp9*	1	1.13	0.85	1.91	0.52	0.95	0.59
*Timp1*	1	0.87	0.25	0.55	0.26	0.39	0.79

**Table 2:** Summary of transcript level changes in oxidative stress, proinflammatory, and tissue injury markers in lung tissue collected from seven mouse strains after subchronic inhalation exposure to acrolein. Numbers indicate the fold change level compared to controls exposed to filtered air.

Mouse strains	Oxidative stress markers					Proinflammatory markers					Tissue injury/repair markers	
	*Gpx1*	*Gpx3*	*Hmox1*	*Nfkb1*	*Sod3*	*Cxcl1*	*Cxcl2*	*IL-6*	*IL-17B*	*Tnfα*	*Mmp9*	*Timp1*
BALB/cByJ	2.3^∗^	2.9^∗^	1.7	2.7^∗^	1.9	2.3^∗^	1.9	2.9^∗^	2.7^∗^	1.7	2.31^∗^	1.1
C57BL/6J	5.6^∗^	3.3^∗^	1.8	2.8^∗^	3.4^∗^	3.5^∗^	2.8^∗^	3.6^∗^	3.9^∗^	2.3^∗^	1.68	2
129S1/SvImJ	1.7	1.6	1.1	2.2^∗^	1.5	2.9^∗^	1.8	4.7^∗^	3.7^∗^	1.8	1.1	1.7
DBA/2J	2.1^∗^	1.8	1.4	0.8	0.9	0.5	1.2	0.7	1.1	0.9	1.9^∗^	3^∗^
C3H/HeJ	2.4	2.8	0.9	1.1	4.7∗	1.4	1.3	1.1	1.5	1.2	2.0^∗^	4.6^∗^
A/J	1.5	2.3	1.3	1.9	0.9	1.5	1.3	2.4^∗^	1.9	2.5	0.9	1.8
FVB/NJ	1.9^∗^	1.9	0.9	0.82	1.2	2.9^∗^	2.9	0.9	1.7	1.1	1.6	3.0^∗^

Glutathione peroxidase 1 (*Gpx1*), *Gpx3*; heme oxygenase 1 (*Hmox1*); *superoxide dismutase 3, extracellular* (*Sod3*); nuclear factor of kappa light polypeptide gene enhancer in B cells 1, p105 (*Nfkb1*); chemokine (C-X-C motif) ligand 1 (*Cxcl1*), Cxcl2 (aka: macrophage inhibitor protein/ *Mip2*); interleukin-6 (*IL-6*), *IL-17B*; tumor necrosis factor alpha (*Tnfα*); *matrix metallopeptidase 9* (*Mmp9*); tissue inhibitor of metalloproteinase 1 (*Timp1*).

## Data Availability

All data required to comprehend the manuscript have been provided in the manuscript's main body and supplementary material. All raw data are available from the corresponding author.

## Supplementary Materials

Supplementary Materials Mouse lung transcript expression analysis following acrolein exposure. Acrolein exposure of the ALI model. Table S1: list of primer pairs used for transcript expression analysis using quantitative real-time polymerase chain reaction. Table S2: bronchoalveolar lavage cell differentials of seven mouse strains after subchronic inhalation exposure to filtered air (sham) and acrolein. Table S3: total concentration of protein in bronchoalveolar lavage of seven mouse strains after subchronic inhalation exposure to filtered air (sham) and acrolein.

## References

[1] Conklin D. J., Malovichko M. V., Zeller I. (2017). Biomarkers of chronic acrolein inhalation exposure in mice: implications for tobacco product-induced toxicity. *Toxicological Sciences*.

[2] Bein (2011). Acrolein - a Pulmonary Hazard. *Molecular Nutrition & Food Research*.

[3] Kasahara D. I., Poynter M. E., Othman Z., Hemenway D., van der Vliet A. (2008). Acrolein inhalation suppresses lipopolysaccharide-induced inflammatory cytokine production but does not affect acute airways neutrophilia. *Journal of Immunology*.

[4] Raju S. V., Jackson P. L., Courville C. A. (2013). Cigarette smoke induces systemic defects in cystic fibrosis transmembrane conductance regulator function. *American Journal of Respiratory and Critical Care Medicine*.

[5] Leikauf G. D., Concel V. J., Liu P. (2011). Haplotype association mapping of acute lung injury in mice implicates activin a receptor, type 1. *American Journal of Respiratory and Critical Care Medicine*.

[6] Borchers M. T., Carty M. P., Leikauf G. D. (1999). Regulation of human airway mucins by acrolein and inflammatory mediators. *The American Journal of Physiology*.

[7] McGuinness A. J. A., Sapey E. (2017). Oxidative stress in COPD: sources, markers, and potential mechanisms. *Journal of Clinical Medicine*.

[8] Bradford M. M. (1976). A rapid and sensitive method for the quantitation of microgram quantities of protein utilizing the principle of protein-dye binding. *Analytical Biochemistry*.

[9] Wesselkamper S. C., Chen L. C., Gordon T. (2001). Development of pulmonary tolerance in mice exposed to zinc oxide fumes. *Toxicological Sciences*.

[10] Dwivedi A. M., Upadhyay S., Johanson G., Ernstgård L., Palmberg L. (2018). Inflammatory effects of acrolein, crotonaldehyde and hexanal vapors on human primary bronchial epithelial cells cultured at air-liquid interface. *Toxicology in Vitro*.

[11] Ji J., Hedelin A., Malmlöf M. (2017). Development of combining of human bronchial mucosa models with Xpose*ALI*® for exposure of air pollution nanoparticles. *PLoS One*.

[12] Thimraj T. A., Sompa S. I., Ganguly K. (2019). Evaluation of diacetyl mediated pulmonary effects in physiologically relevant air-liquid interface models of human primary bronchial epithelial cells. *Toxicology in Vitro*.

[13] Strandberg K., Palmberg L., Larsson K. (2008). Effect of budesonide and formoterol on Il-6 and Il-8 release from primary bronchial epithelial cells. *The Journal of Asthma*.

[14] von Scheele I., Larsson K., Palmberg L. (2010). Budesonide enhances toll-like receptor 2 expression in activated bronchial epithelial cells. *Inhalation Toxicology*.

[15] Zhu S., Qian Y. (2012). Il-17/Il-17 receptor system in autoimmune disease: mechanisms and therapeutic potential. *Clinical science*.

[16] Li L., Holian A. (1998). Acrolein: a respiratory toxin that suppresses pulmonary host defense. *Reviews on environmental health*.

[17] Rahman I. (2002). Oxidative stress and gene transcription in asthma and chronic obstructive pulmonary disease: antioxidant therapeutic targets. *Current Drug Targets. Inflammation and Allergy*.

[18] Rahman I., Marwick J., Kirkham P. (2004). Redox modulation of chromatin remodeling: impact on histone acetylation and deacetylation, NF-*κ*B and pro-inflammatory gene expression. *Biochemical Pharmacology*.

[19] vanden Berghe W., de Bosscher K., Boone E., Plaisance S., Haegeman G. (1999). The nuclear Factor-*κ*B engages Cbp/P300 and histone acetyltransferase activity for transcriptional activation of the interleukin-6 gene promoter. *Journal of Biological Chemistry*.

[20] Sheller J. R., Polosukhin V. V., Mitchell D., Cheng D.-S., Peebles R. S., Blackwell T. S. (2009). Nuclear factor kappa B induction in airway epithelium increases lung inflammation in allergen-challenged mice. *Experimental Lung Research*.

[21] Lingappan K. (2018). NF-*κ*B in oxidative stress. *Current opinion in toxicology*.

[22] Cheng D.-s., Han W., Chen S. M. (2007). Airway epithelium controls lung inflammation and injury through the Nf-kappa B pathway. *The Journal of Immunology*.

[23] Libermann T. A., Baltimore D. (1990). Activation of interleukin-6 gene expression through the NF-kappa B transcription factor. *Molecular and cellular biology*.

[24] Liu T., Zhang L., Joo D., Sun S.-C. (2017). NF-*κ*B signaling in inflammation. *Signal Transduction and Targeted Therapy*.

[25] Deshmukh H. S., Shaver C., Case L. M. (2008). Acrolein-activated matrix metalloproteinase 9 contributes to persistent mucin production. *American journal of respiratory cell and molecular biology*.

[26] Shapiro S. D., Ingenito E. P. (2005). The pathogenesis of chronic obstructive pulmonary disease: advances in the past 100 years. *American Journal of Respiratory Cell and Molecular Biology*.

[27] Johnatty R. N., Taub D. D., Reeder S. P. (1997). Cytokine and chemokine regulation of Prommp-9 and Timp-1 production by human peripheral blood lymphocytes. *The Journal of Immunology*.

[28] Kossakowska A. E., Edwards D. R., Prusinkiewicz C. (1999). Interleukin-6 regulation of matrix metalloproteinase (Mmp-2 and Mmp-9) and tissue inhibitor of metalloproteinase (Timp-1) expression in malignant non-Hodgkin’s lymphomas. *Blood*.

[29] Lindén A., Dahlén B. (2014). Interleukin-17 cytokine signalling in patients with asthma. *The European Respiratory Journal*.

[30] Wang Y.-H., Wills-Karp M. (2011). The Potential Role of Il-17 in Severe Asthma. *Current Allergy and Asthma Reports*.

[31] Bozinovski S., Seow H. J., Chan S. P. J. (2015). Innate cellular sources of interleukin-17a regulate macrophage accumulation in cigarette- smoke-induced lung inflammation in mice. *Clinical Science (London, England)*.

[32] Strzelak A., Ratajczak A., Adamiec A., Feleszko W. (2018). Tobacco smoke induces and alters immune responses in the lung triggering inflammation, allergy, asthma and other lung diseases: a mechanistic review. *International journal of environmental research and public health*.

