# Supplementary material for: Analysis of Acrolein Exposure Induced Pulmonary Response in Seven Inbred Mouse Strains and Human Primary Bronchial Epithelial Cells Cultured at Air-Liquid Interface
Source: Biomed Res Int. 2020 Oct 8;2020:3259723. doi: 10.1155/2020/3259723 (PMC7582059; doi:10.1155/2020/3259723)
Supplement: Supplementary Materials — Mouse lung transcript expression analysis following acrolein exposure. Acrolein exposure of the ALI model. Table S1: list of primer pairs used for transcript expression analysis using quantitative real-time polymerase chain reaction. Table S2: bronchoalveolar lavage cell differentials of seven mouse strains after subchronic inhalation exposure to filtered air (sham) and acrolein. Table S3: total concentration of protein in bronchoalveolar lavage of seven mouse strains after subchronic inhalation exposure to filtered air (sham) and acrolein. [file 3259723.f1.docx]

**Supplementary data**

**Acrolein exposure of inbred mouse strains and human primary bronchial epithelial cells cultured at air-liquid interface identifies interleukin 17 pathway as a plausible target**

Gunnar Johanson^1^, Aishwarya Mishra Dwivedi^1^, Lena Ernstgård^1^, Lena Palmberg^1^, Koustav Ganguly^1^, Lung Chi Chen^2^, Karen Galdanes ^2^, Terry Gordon^2^, Swapna Upadhyay^1*^,

1: Integrative Toxicology, Institute of Environmental Medicine, Karolinska Institute, Stockholm, Sweden.

2: Department of Environmental Medicine, NYU School of Medicine, NY, USA

| Gunnar Johanson | Gunnar.Johanson@ki.se |
| --- | --- |
| Aishwarya Mishra Dwivedi | aishmeister@gmail.com |
| Lena Ernstgård | Lena.Ernstgård@ki.se |
| Lena Palmberg | lena.palmberg@ki.se |
| Koustav Ganguly | koustav.ganguly@ki.se |
| Lung Chi Chen | [lung-chi.chen@nyumc.org](mailto:lung-chi.chen@nyumc.org) |
| Karen Galdanes | karengaldanes@gmail.com |
| Terry Gordon | Terry.Gordon@nyumc.org |
| Swapna Upadhyay | swapna.upadhyay@ki.se |

*Correspondence:

Swapna Upadhyay

Institute of Environmental Medicine

Karolinska Institutet

Nobels väg 13, Box 210, SE-171 77 Stockholm, Sweden

Phone: +46 852487930

Email: [swapna.upadhyay@ki.se](mailto:swapna.upadhyay@ki.se)

### Mice lung transcript expression analysis following acrolein exposure: Total lung RNA was isolated using the RNeasy kit (#74104; Qiagen, Hilden, Germany) according to manufacturer’s instruction and the concentration was quantified using Nanodrop 2100 (ThermoFisher, USA). Extracted total RNA was stored at -80°C until further use. A High Capacity RNA-to-cDNA kit (AB Applied Biosystems) was used for reverse transcription to produce cDNA. Quantitative real-time polymerase chain reaction (qRT-PCR) was performed using Applied Biosystem instrument (ABI 7500, USA) and SYBR Green reagents according to manufacturer’s protocol (ThermoFisher, USA). qRT-PCR was performed, and the comparative cycle number threshold (C_T_) method (ΔΔC_T_) was used [ΔC_T_=C_T_ (gene)-C_T_(*Actb*)] to calculate the fold changes. Data are presented as the transcript expression in exposed animals relative to strain specific control level median and 25^th^-75^th^ percentiles; n=5 mice/strain/group,. Transcripts assayed using the qRT-PCR technique included: beta actin (*Actb*), glutathione peroxidase 1 and 3 (*Gpx1, Gpx3);* heme oxygenase 1 (*Hmox1), superoxide dismutase 3, extracellular (Sod3),* nuclear factor of kappa light polypeptide gene enhancer in B cells 1, p105 (*Nfkb1),* tumor necrosis factor (*Tnf)* chemokine (C-X-C motif) ligand 1 (*Cxcl1),* *Cxcl2, interleukin 6 (Il6), Il17b,* matrix metalloproteinase 9 *(Mmp9*), tissue inhibitor of metalloproteinase 1 (*Timp1*). Beta actin (*Actb*) was used as the reference control. Forward and reverse primer sequences are provided in Table 1.

**Acrolein exposure of ALI model:**

Human PBECs cultured in a physiologically relevant ALI were exposed clean air or to 0.1 and 0.2 ppm acrolein vapor for 30 minutes using our in-house developed exposure systems as described in our previous study by (Dwivedi et al, 2018, Thimraj et al., 2019). Clean air exposed samples served as the sham control. Transcript expression of *IL17A, IL17B, IL17 C, IL17D, IL17E, IL1B, IL22*, RAR related orphan receptor A (*RORA*), and signal transducer and activator of transcription 3 (*STAT3*) were analyzed following 24h of incubation after 30 minutes exposure to to clean air and acrolein. Beta actin (Actb) was used as the reference control. Forward and reverse primer sequences are provided in SupplementaryTable 1.

BAL analysis: Total BAL cell counts, and protein concentrations showed no changes in the acrolein exposed mice compared to their respective strain control exposure (supplementary Table 2 and Table 3).

References:

Dwivedi et al 2018. Inflammatory effects of acrolein, crotonaldehyde and hexanal vapors on human primary bronchial epithelial cells cultured at air-liquid interface. Toxicol In Vitro. Feb;, 46, 219-228.

Thimraj et al. 2019. Evaluation of diacetyl mediated pulmonary effects in physiologically relevant air-liquid interface models of human primary bronchial epithelial cells. *Toxicol In Vitro,* 61:104617

**Table S1:** List of primer pairs used for transcript expression analysis using quantitative real-time polymerase chain reaction (qRT-PCR).

| **Gene symbol** | **Gene name** | **Forward Primer (5´-3´)** | **Reverse Primer (3´-5´)** |
| --- | --- | --- | --- |
| **Mouse** |  |  |  |
| *Actb* | Beta actin | GGTGGGAATGAGAAGG | GGGGTACTTCTCAGGA |
| *Gpx1* | Glutathione peroxidase 1 | GGAAAGAAAGCGATGCCACGT | GAAGGCATACACGGTGGACTGTG |
| *Gpx3* | Glutathione peroxidase 3 | GCATACCGGTTATGCGCTGGTA | TGGGAATGTGGCTTCCTTCCTG |
| *Ho1* | Heme oxygenase 1 | AGATTGCACAGAAGGCCATGGC | CAATGTTGAGCAGGAAGGCGGT |
| *Sod3* | Superoxide dismutase 3 | CTTGGTTCTCTTCCGGCAGCTG | ACCACGAAGTTGCCAAAGTCGC |
| *Nfkb1* | Nuclear factor of kappa light polypeptide gene enhancer in B cells 1 | GAGGTCTCTGTACCA | TGTAAAATGCGGGGAA |
| *Cxcl1* | Chemokine (C-X-C motifligand 1) | ACCGAAGTCACACACT | GTGCCATCAGGTCTGT |
| *Cxcl2* | Chemokine (C-X-C motif ligand 2) | CTCTCAAGGGCGGTCAAAAAGTT | TCAGACAGCGAGGCACATCAGGTA |
| *Tnf* | Tumor necrosis factor | TAGCCCACGCAAAC | ACCCTGAGCCTCCCCT |
| *Il17b* | Interleukin 17B | ATGGGGCTACTCAACC | CGCAACCCAAAGGCAC |
| *Il6* | Interleukin 6 | AGTCTCCTCTACTTGT | TTCCTCTCTGACTTCC |
| *Mmp9* | Matrix metallopeptidase 9 | TGAAGACTTGCCGCGAGACATG | AACTCGTCGTCGTCGAAATGGG |
| *Timp1* | Tissue inhibitor of metalloproteinase 1 | GCGGTGGGTGGATGAGTAATGC | GGATGCCAGATGCCAGAGATGC |
| **Human** |  |  |  |
| IL17A | Interleukin 17A | CCCGGACTGTGATGGTCAAC | GCTCTTCACAGTGGTCCTTCC |
| IL17B | Interleukin 17B | GCACTGCCCTCATTGTTCTTC | TGGAGGTTTGGATTGGGCAT |
| *IL17C* | Interleukin 17C | GTACCCCACACTGCTACTCG | CCAGGTCCTCACCGGTATCT |
| *IL17D* | Interleukin 17D | CCTTCGGGTTATTCCGCTCA | GACTCTGCACCTTTCGCCT |
| *IL17F* | Interleukin 17F | TGCTAGTAGGTGAGGCGAGT | AATCAGTCTCATTTGCACTTACG |
| *IL1B* | Interleukin 1 beta | CCA GCT ACG AAT CTC CGA CC | AGA ACA CCA CTT GTT GCT CCA |
| *IL22* | Interleukin 22 | CTCCATGGGTGTGGAGGTTC | CCAAAGTAATCGCCCTGGTG |
| RORA | RAR related orphan receptor A alpha | CAGCTGGGCAAATACACTACG | ATGCACTTGGTGGAGAGTTCA |
| (STAT3) | Signal transducer and activator of transcription 3 | TGGAAGAGGCGGCAGCAGATAGC | CACGGCCCCCATTCCCACAT |

Table S2: Bronchoalveolar lavage cell differentials seven mouse strains after subchronic inhalation exposure to filtered air (sham) and acrolein.

| **Mice Strain** | **Macrophage**  **(% of total cells)** | | **PMN**  **(% of total cells)** | | **Epithelial cells**  **(% of total cells)** | |
| --- | --- | --- | --- | --- | --- | --- |
|  | **Sham** | **Acrolein** | **Sham** | **Acrolein** | **Sham** | **Acrolein** |
| BALB/cByJ | 94.3±1.0 | 92.6±0.8 | 0.75±0.5 | 1.2±0.42 | 5.1±1.5 | 6.2±0.6 |
| C57BL6J | 92.0±1.2 | 92.2±0.7 | 0.5±0.4 | 0.8±0.42 | 7.5±1.6 | 7±1.04 |
| DBA/2J | 87.8±3.1 | 90.2±2.6 | 3.8±1.2 | 0.2±0.2 | 8.4±2.0 | 9.6±2.4 |
| FVB/NJ | 92.0±2.5 | 89.0±3.4 | 1.0±1.1 | 0.6±0.4 | 7.0±2.8 | 10.2±3.2 |
| A/J | 84.2±2.6 | 90.6±4.0 | 4.4±3.7 | 0.2±0.2 | 9.6±3.8 | 9.2±4.0 |
| 129S1/SvImJ | 89.0±2.2 | 82.0±8.25 | 6.0±6.2 | 13.4±8.2 | 6.8±2.3 | 4.8±1.33 |
| C3H/HeJ | 84.0±6.7 | 78.2±8.7 | 8.4±4.7 | 20.8±11.1 | 7.2±2.0 | 7.4±0.9 |

Table S3: Total concentration of protein in bronchoalveolar lavage of seven mouse strains after subchronic inhalation exposure to filtered air (sham) and acrolein.

| **Mice Strains** | **Protein Concentration (µg/ml)** | |
| --- | --- | --- |
|  | **Sham** | **Acrolein** |
| BALB/cByJ | 328.0±29.6 | 376.5±24.5 |
| C57BL6J | 352.5±57.4 | 232.2±39.6 |
| DBA/2J | 425.3±58.9 | 317.6±23.2 |
| FVB7NJ | 443.3±47.2 | 390.0±33.8 |
| A/J | 361.9±72.7 | 279.7±52.4 |
| 129S1/SvImJ | 246.4±21.2 | 274.8±39.2 |
| C3H/HeJ | 171.3±8.8 | 205.7±23.3 |
